# Association between wet-bulb globe temperature and kidney function in different geographic regions in a large Taiwanese population study

**DOI:** 10.1093/ckj/sfae173

**Published:** 2024-06-22

**Authors:** Wei-Yu Su, Ping-Hsun Wu, Ming-Yen Lin, Pei-Yu Wu, Yi-Chun Tsai, Yi-Wen Chiu, Jer-Ming Chang, Chih-Hsing Hung, Chih-Da Wu, Chao-Hung Kuo, Szu-Chia Chen

**Affiliations:** Graduate Institute of Clinical Medicine, College of Medicine, Kaohsiung Medical University, Kaohsiung, Taiwan; Department of General Medicine, Kaohsiung Medical University Hospital, Kaohsiung Medical University, Kaohsiung, Taiwan; Division of Nephrology, Department of Internal Medicine, Kaohsiung Medical University Hospital, Kaohsiung Medical University, Kaohsiung, Taiwan; Department of General Medicine, Kaohsiung Medical University Hospital, Kaohsiung Medical University, Kaohsiung, Taiwan; Division of Nephrology, Department of Internal Medicine, Kaohsiung Medical University Hospital, Kaohsiung Medical University, Kaohsiung, Taiwan; Faculty of Medicine, College of Medicine, Kaohsiung Medical University, Kaohsiung, Taiwan; Division of Nephrology, Department of Internal Medicine, Kaohsiung Medical University Hospital, Kaohsiung Medical University, Kaohsiung, Taiwan; Graduate Institute of Clinical Medicine, College of Medicine, Kaohsiung Medical University, Kaohsiung, Taiwan; Division of Nephrology, Department of Internal Medicine, Kaohsiung Medical University Hospital, Kaohsiung Medical University, Kaohsiung, Taiwan; Faculty of Medicine, College of Medicine, Kaohsiung Medical University, Kaohsiung, Taiwan; Department of Internal Medicine, Kaohsiung Municipal Siaogang Hospital, Kaohsiung Medical University, Kaohsiung, Taiwan; Division of Nephrology, Department of Internal Medicine, Kaohsiung Medical University Hospital, Kaohsiung Medical University, Kaohsiung, Taiwan; Faculty of Medicine, College of Medicine, Kaohsiung Medical University, Kaohsiung, Taiwan; Division of Nephrology, Department of Internal Medicine, Kaohsiung Medical University Hospital, Kaohsiung Medical University, Kaohsiung, Taiwan; Faculty of Medicine, College of Medicine, Kaohsiung Medical University, Kaohsiung, Taiwan; Division of Nephrology, Department of Internal Medicine, Kaohsiung Medical University Hospital, Kaohsiung Medical University, Kaohsiung, Taiwan; Faculty of Medicine, College of Medicine, Kaohsiung Medical University, Kaohsiung, Taiwan; Research Center for Precision Environmental Medicine, Kaohsiung Medical University, Kaohsiung, Taiwan; Department of Pediatrics, Kaohsiung Medical University Hospital, Kaohsiung Medical University, Kaohsiung, Taiwan; Department of Pediatrics, Kaohsiung Municipal Siaogang Hospital, Kaohsiung Medical University, Kaohsiung, Taiwan; Research Center for Precision Environmental Medicine, Kaohsiung Medical University, Kaohsiung, Taiwan; Department of Geomatics, National Cheng Kung University, Tainan, Taiwan; National Institute of Environmental Health Sciences, National Health Research Institutes, Miaoli, Taiwan; Innovation and Development Center of Sustainable Agriculture, National Chung Hsing University, Taichung, Taiwan; Faculty of Medicine, College of Medicine, Kaohsiung Medical University, Kaohsiung, Taiwan; Department of Internal Medicine, Kaohsiung Municipal Siaogang Hospital, Kaohsiung Medical University, Kaohsiung, Taiwan; Division of Gastroenterology, Department of Internal Medicine, Kaohsiung Medical University Hospital, Kaohsiung Medical University, Kaohsiung, Taiwan; Division of Nephrology, Department of Internal Medicine, Kaohsiung Medical University Hospital, Kaohsiung Medical University, Kaohsiung, Taiwan; Faculty of Medicine, College of Medicine, Kaohsiung Medical University, Kaohsiung, Taiwan; Department of Internal Medicine, Kaohsiung Municipal Siaogang Hospital, Kaohsiung Medical University, Kaohsiung, Taiwan; Research Center for Precision Environmental Medicine, Kaohsiung Medical University, Kaohsiung, Taiwan

**Keywords:** different geographic region, estimated glomerular filtration rate, Taiwan Biobank, wet-bulb globe temperature

## Abstract

The worldwide prevalence and incidence rates of end-stage renal disease have been increasing, and the trend is pronounced in Taiwan. This is especially evident in southern Taiwan, where the wet-bulb globe temperature (WBGT) is consistently higher than in other regions. The association between kidney function and WBGT has not been fully investigated. Therefore, the aim of this study was to evaluate the association between estimated glomerular filtration rate (eGFR) and WBGT and variations in this association across different geographic regions in Taiwan. We used the Taiwan Biobank (TWB) to obtain data on community-dwelling individuals, linked these data with WBGT data obtained from the Central Weather Bureau and then processed the data using a machine learning model. WBGT data were recorded during the working period of the day from 8:00 a.m. to 5:00 p.m. These data were then compiled as 1-year, 3-year and 5-year averages, recorded prior to the survey year of the TWB of each participant. We identified 114 483 participants who had WBGT data during 2012–2020. Multivariable analysis showed that, in northern Taiwan, increases in 1- and 3-year averages of WBGT during the working period (β = −0.092, *P* = .043 and β = −0.193, *P* < .001, respectively) were significantly associated with low eGFR. In southern Taiwan, increases in 1-, 3- and 5-year averages of WBGT during the working period (β = −0.518, *P* < .001; β = −0.690, *P* < .001; and β = −0.386, *P* = .001, respectively) were gnificantly associated with low eGFR. These findings highlight the importance of heat protection for people working outdoors or in high-temperature environments as a measure to prevent negative impacts on kidney function. Moreover, we observed that in southern Taiwan, every 1°C increase in WBGT had a greater impact on the decrease in eGFR compared with other regions in Taiwan.

## Graphical Abstract

**Graphical Abstract:**
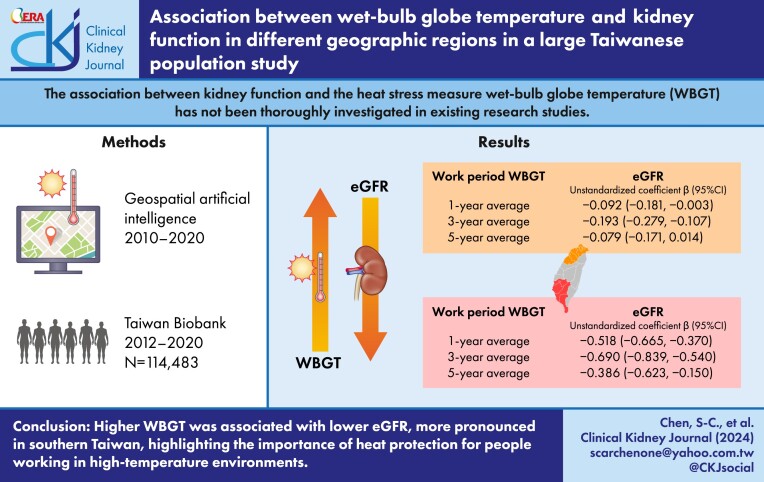

KEY LEARNING POINTS **What was known:**

- The global prevalence and incidence of end-stage renal disease are increasing, with Taiwan, especially southern Taiwan, experiencing pronounced increases due to higher wet-bulb globe temperatures (WBGTs).

**This study adds:**

- We demonstrated a significant association between higher WBGTs and lower estimated glomerular filtration rate (eGFR) in Taiwan.
- We revealed that increases in WBGT significantly correlate with reduced kidney function, with a greater impact observed in southern Taiwan.

**Potential impact:**

- This research underlines the necessity for heat protection measures for individuals working outdoors or in high-temperature settings, especially in regions with higher WBGTs.
- It may influence public health policies and workplace regulations to mitigate the risks of kidney health deterioration due to exposure to high temperatures.

## INTRODUCTION

The worldwide prevalence and incidence rates of end-stage renal disease (ESRD) have been steadily increasing over the years, and the trend is particularly pronounced in Taiwan [1]. However, regional differences in the incidence rate of ESRD have been reported, with a recent study showing higher incidence rates of ESRD in the southern and southeastern regions of Taiwan than in other areas [2]. Several potential causes have been proposed, including socio-environmental factors such as the proportion of elderly and Aboriginal people, healthcare resources, unemployment rates and education levels [2].

Since 1970, the global average temperature has notably increased, leading not only to significant environmental changes, such as the shrinking of mountain glaciers, rising sea levels and alterations in the biosphere [3], but also to negative impacts on human health [4]. The increasing temperature has been associated with increases in cardiovascular [5], respiratory [6] and renal [7] diseases, and even mortality [8]. The increase in ambient temperature has also been linked to a decline in kidney function, including both acute and chronic impacts [9]. A similar situation can be seen in the model of Mesoamerican nephropathy, which is characterized by kidney injury with chronic tubulointerstitial disease due to exposure to high temperatures and humidity [7]. One of the potential mechanisms links hyperosmolarity with the activation of vasopressin, as well as the aldose reductase–fructokinase pathway [7]. Compared with ambient temperature, the wet-bulb globe temperature (WBGT), which was developed and first used in US military training to reduce severe heat-related illness [10], integrates temperature and humidity to more accurately reflect the impact of heat stress on health, particularly in active populations outdoors [11]. The WBGT in southern Taiwan has been reported to be higher than in other regions of Taiwan, both in terms of annual average and across all four seasons [12]. This distinct regional characteristic led us to consider whether WBGT may be a contributing factor to the higher incidence of ESRD in southern regions. In addition, although previous research has reported a significant association between high temperatures and kidney disease [9, 13], few studies have considered the combined impact of temperature and humidity on kidney function.

In this study, we used the Taiwan Biobank (TWB) to obtain data on community-dwelling individuals and linked these data with the WBGT. The WBGT was calculated using temperature, humidity and solar radiation data obtained from the Central Weather Bureau and then processed through a machine learning model. The objectives of the study were to evaluate the association between WBGT and estimated glomerular filtration rate (eGFR) and to explore variations in this association across different geographic regions in Taiwan.

## MATERIALS AND METHODS

### Subject recruitment from the TWB

The TWB, initiated by Taiwan's Ministry of Health and Welfare, aims to enhance healthcare services, mitigate chronic diseases and tackle challenges related to the aging population. This comprehensive resource compiles medical, genetic and lifestyle information from cancer-free individuals, 30–70 years of age, drawn from various communities across Taiwan [14, 15]. The TWB's establishment and operations received ethical endorsements from both the Ethics and Governance Council of the TWB and Institutional Review Board on Biomedical Science Research at Academia Sinica in Taiwan.

Upon consent for participation in the TWB, data are acquired from the participants using a systematic protocol. This protocol encompasses structured in-person interviews, physical examinations and the collection of blood specimens. During this enrolment phase, data including body height, weight, body mass index (BMI; kg/m^2^), age, sex, smoking and alcohol consumption, medical history [including hypertension and diabetes mellitus (DM)] are compiled. The protocol extends to an array of laboratory assessments, including fasting glucose levels, haemoglobin, triglycerides, total cholesterol, high-density lipoprotein (HDL) cholesterol, low-density lipoprotein (LDL) cholesterol, eGFR (calculated using the 2021 Chronic Kidney Disease Epidemiology Collaboration creatinine equation) [16] and uric acid.

Blood pressure (BP) readings are acquired digitally by trained personnel. Prior to the measurement, the participants are instructed to abstain from caffeine, physical exercise and smoking for a minimum of 30 minutes. The BP measurement protocol involves three consecutive readings, each separated by an interval of 1–2 minutes. For the purposes of this study, the average values of systolic and diastolic BP were utilized in the analyses. Regular exercise was defined as engaging in physical activity for a duration of at least 30 minutes a minimum of three times per week. The study's methodology adhered to the principles outlined in the Declaration of Helsinki and received ethical approval from the Institutional Review Board of Kaohsiung Medical University Hospital [KMUHIRB-E(I)-20210058].

### Study participants

We identified 115 423 participants in the TWB and excluded those without data for WBGT due to residing offshore (*n* = 940). The remaining 114 483 participants were enrolled, all of whom provided written informed consent.

**Figure 1:**
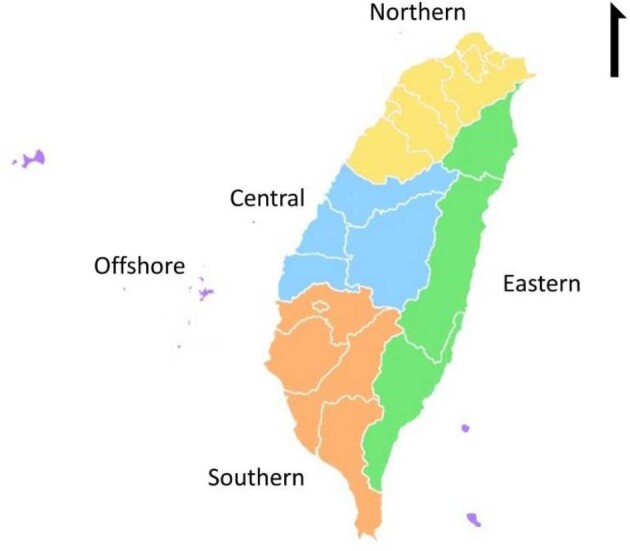
The distribution of different regions in Taiwan.

The participants were geographically distributed across four major areas of Taiwan: the northern, central, southern and eastern regions (Fig. 1). These areas, while serving as administrative divisions, also exhibit slight climatic differences. The southern region, comprising five cities/counties, has a tropical climate. In contrast, the central and northern regions, comprising four and seven cities/counties, respectively, are characterized by a subtropical climate. The eastern region of Taiwan, encompassing five counties and distinguished by its slender north-to-south stretch, covers areas within both subtropical and tropical climatic zones.

### Assessment of WBGT

The study utilized hourly temperature data from 453 weather stations in Taiwan managed by the Central Weather Bureau [12]. This dataset, spanning from 2000 to 2020, included records from 427 automatic and 26 manual stations. The WBGT at each monitoring station was calculated using temperature, humidity and solar radiation data with the following equation: WBGT = 0.7*t*_nw_ + 0.2*t*_g_ + 0.1*t*_a_, where *t*_nw_ is the natural wet-bulb temperature, *t*_g_ is the globe temperature and *t*_a_ is the dry-bulb temperature. The WBGT values were then categorized into two distinct exposure windows: the working period, spanning from 8:00 a.m. to 5:00 p.m., and the noon period, between 11:00 a.m. and 2:00 p.m. These hourly WBGT values were then used as the dependent variable for developing a land use–based spatial machine learning (LBSM) model for predicting highly spatial-temporal variations of WBGT on the main island of Taiwan. Factors that could affect WBGT, including weather conditions such as relative humidity, wind speed, rainfall and solar declination, were also collected. Land use information encompassing parks, water, industrial and residential areas, along with road network details and points of interest such as Chinese restaurants and temples, was also gathered. SHapley Additive exPlanation (SHAP) values were used as a criterion for important predictor selection. A light gradient boosting machine (LightGBM) algorithm coupled with the selected predictor variables was then used to build the prediction model. Incorporating temperature data with other land use/land cover predictor variables significantly enhanced the performance of the LBSM model, achieving an *R*^2^ value as high as 0.99 [17]. Using the LBSM model, we obtained spatial-temporal WBGT levels at a high-resolution grid of 50 m × 50 m.

### Linking data from the TWB and WBGT

Data from the TWB were linked to our WBGT data by residential area at the township level, based on the participant's residential address, to estimate the participant's heat exposure. The average annual WBGT exposure was estimated. For each participant, the average WBGT levels for 1, 3 and 5 years preceding the survey year of TWB were recorded. These WBGT levels were used as indicators of both short-term and long-term environmental exposure in this study.

### Statistical analysis

Statistical analysis was performed using SPSS version 25 for Windows (IBM, Armonk, NY, USA). Data were expressed as percentage or mean ± standard deviation (SD). Linear regression analysis was used to assess the association between WBGT and eGFR. Significant variables in univariable analysis were then analysed using multivariable analysis. *P*-values <.05 were considered statistically significant.

## RESULTS

### Baseline clinical characteristics of all participants

The baseline clinical characteristics of the participants are shown in Table 1. The mean age of the 114 483 participants was 50.0 ± 11.0 years and included 41 141 males and 73 342 females. The participants in the study were distributed across Taiwan, with 35.1% residing in northern Taiwan, 20.1% in central Taiwan, 34.8% in southern Taiwan and 10% in eastern Taiwan. The average WBGT values during the noon period at 1, 3 and 5 years prior to the survey year were 27.3 ± 1.1, 27.1 ± 1.1 and 27.0 ± 1.1°C, respectively. For the working period, the corresponding average WBGT values were 25.0 ± 1.2, 24.9 ± 1.2 and 24.9 ± 1.2°C, respectively.

**Table 1:** Baseline clinical characteristics among participants.

Characteristics	Values (*N* = 114 483)
Age (years), mean ± SD	50.0 ± 11.0
Male, %	35.9
Smoking history, %	27.5
Alcohol history, %	8.5
DM, %	9.6
Hypertension, %	24.9
BMI (kg/m^2^), mean ± SD	24.2 ± 3.8
Systolic BP (mmHg), mean ± SD	120.8 ± 18.6
Diastolic BP (mmHg), mean ± SD	74.0 ± 11.4
Regular exercise habits, %	40.6
Laboratory parameters, mean ± SD	
Fasting glucose (mg/dl)	95.9 ± 20.7
Triglyceride (mg/dl)	115.4 ± 94.0
Total cholesterol (mg/dl)	195.9 ± 35.9
HDL-cholesterol (mg/dl)	54.5 ± 13.5
LDL-cholesterol (mg/dl)	121.0 ± 31.8
Haemoglobin (g/dl)	13.8 ± 1.6
Uric acid (mg/dl)	5.4 ± 1.4
eGFR (ml/min/1.73 m^2^)	105.4 ± 13.8
eGFR <60 ml/min/1.73 m^2^ (%)	0.9
Residence, %	
Northern Taiwan	35.1
Central Taiwan	20.1
Southern Taiwan	34.8
Eastern Taiwan	10.0
Average of WBGT during the noon period^a^ (per 1°C)	
1-year	27.3 ± 1.1
3-year	27.1 ± 1.1
5-year	27.0 ± 1.1
Average of WBGT during the work period^a^ (per 1°C)	
1-year	25.0 ± 1.2
3-year	24.9 ± 1.2
5-year	24.9 ± 1.2

a The noon period is defined as 11 a.m. to 2 p.m. and the work period is defined as 8 a.m. to 5 p.m.

### Determinants of eGFR using univariable linear regression analysis

Univariable linear regression analysis was conducted to explore the determinants of eGFR. The results revealed that older age, male sex, smoking and alcohol history, DM, hypertension, high BMI, elevated systolic and diastolic BPs, regular exercise, increased fasting glucose, high triglycerides, elevated total cholesterol, low HDL cholesterol, high LDL cholesterol, increased haemoglobin and high uric acid levels were all significantly correlated with lower eGFR (Table 2).

**Table 2:** Determinants for eGFR using univariable linear regression analysis.

Parameters	eGFR (univariable), unstandardized coefficient β (95% CI)	*P*-value
Age (per 1 year)	−0.776 (−0.782 to −0.771)	<.001
Male (versus female)	−7.255 (−7.416 to −7.093)	<.001
Smoking history	−3.489 (−3.667 to −3.311)	<.001
Alcohol history	−3.856 (−4.142 to −3.571)	<.001
DM	−7.200 (−7.469 to −6.931)	<.001
Hypertension	−9.415 (−9.592 to −9.238)	<.001
BMI (per 1 kg/m^2^)	−0.452 (−0.473 to −0.431)	<.001
Systolic BP (per 1 mmHg)	−0.243 (−0.247 to −0.239)	<.001
Diastolic BP (per 1 mmHg)	−0.271 (−0.278 to −0.264)	<.001
Regular exercise habits	−6.476 (−6.635 to −6.317)	<.001
Laboratory parameters		
Fasting glucose (per 1 mg/dl)	−0.102 (−0.106 to −0.098)	<.001
Triglyceride (per 100 mg/dl)	−0.021 (−0.021 to −0.020)	<.001
Total cholesterol (per 1 mg/dl)	−0.049 (−0.051 to −0.046)	<.001
HDL cholesterol (per 1 mg/dl)	0.115 (0.109–0.121)	<.001
LDL cholesterol (per 1 mg/dl)	−0.045 (−0.047 to −0.042)	<.001
Haemoglobin (per 1 g/dl)	−1.641 (−1.690 to −1.592)	<.001
Uric acid (per 1 mg/dl)	−3.484 (−3.536 to −3.432)	<.001

### Association between WBGT and eGFR

Table 3 illustrates that average WBGT values for both the noon and working periods at 1, 3 and 5 years were significantly negatively associated with eGFR using multivariable linear regression analysis after adjusting for age, sex, smoking and alcohol history, DM, hypertension, BMI, systolic and diastolic BPs, regular exercise, fasting glucose, triglycerides, total cholesterol, HDL cholesterol, LDL cholesterol, haemoglobin and uric acid (the significant variables listed in Table 2). During the noon period, the 1-year average WBGT (per 1°C; unstandardized coefficient β = −0.141; *P* < .001), 3-year average WBGT (per 1°C; unstandardized coefficient β = −0.196; *P* < .001) and 5-year average WBGT (per 1°C; unstandardized coefficient β = −0.156; *P* < .001) values were significantly negatively associated with eGFR. Similarly, during the working period, 1-year average WBGT (per 1°C; unstandardized coefficient β = −0.053; *P* = .030), 3-year average WBGT (per 1°C; unstandardized coefficient β = −0.117; *P* < .001) and 5-year average WBGT (per 1°C; unstandardized coefficient β = −0.064; *P* = .017) values were significantly negatively associated with eGFR.

**Table 3:** Association of WBGT with eGFR using multivariable linear regression analysis.

Average of WBGT (per 1°C)	eGFR (multivariable), unstandardized coefficient β (95% CI)	*P*-value
Noon period		
1-year	−0.141 (−0.193 to −0.090)	<.001
3-year	−0.196 (−0.247 to −0.145)	<.001
5-year	−0.156 (−0.212 to −0.101)	<.001
Work period		
1-year	−0.053 (−0.101 to −0.005)	.030
3-year	−0.117 (−0.165 to −0.069)	<.001
5-year	−0.064 (−0.117 to −0.011)	.017

Multivariable model: adjusted for age, sex, smoking and alcohol history, diabetes, hypertension, BMI, systolic and diastolic BPs, regular exercise, fasting glucose, triglycerides, total cholesterol, HDL cholesterol, LDL cholesterol, haemoglobin and uric acid.

### Association between WBGT and eGFR in different geographic regions

**Figure 2:**
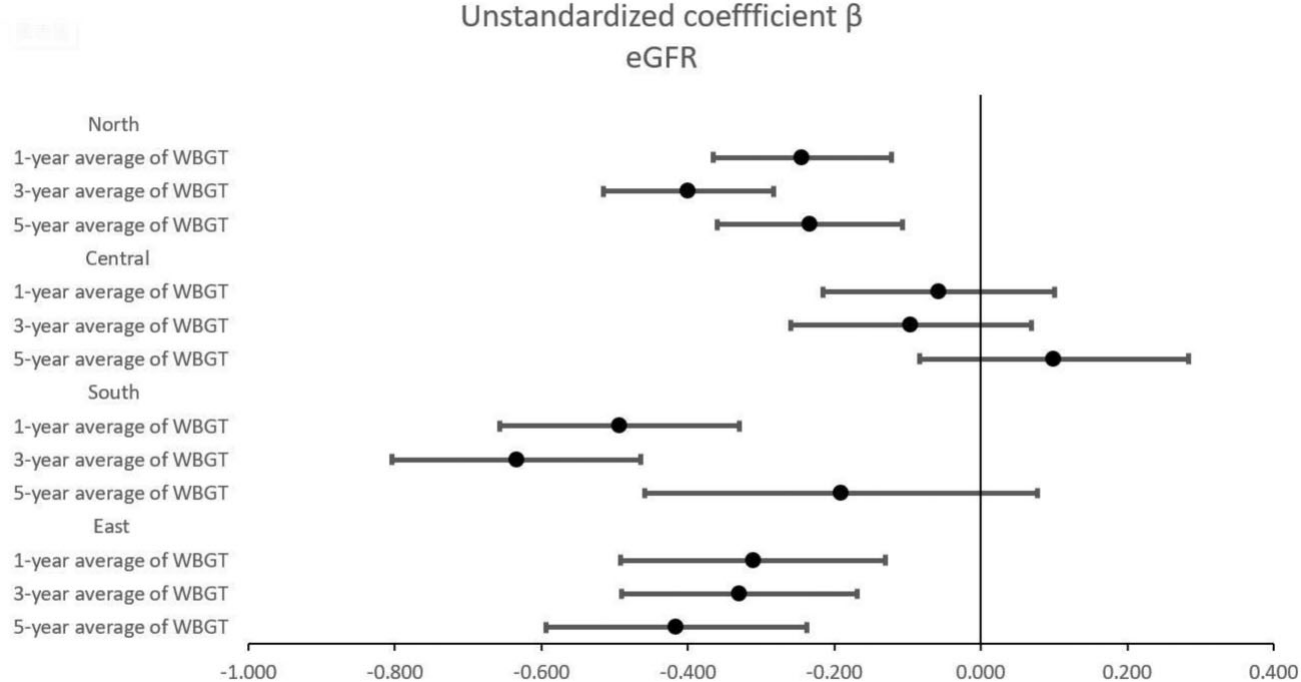
Forest plots of the association of WBGT in the noon period with eGFR using multivariable linear regression analysis stratification by geographic regions.

**Figure 3:**
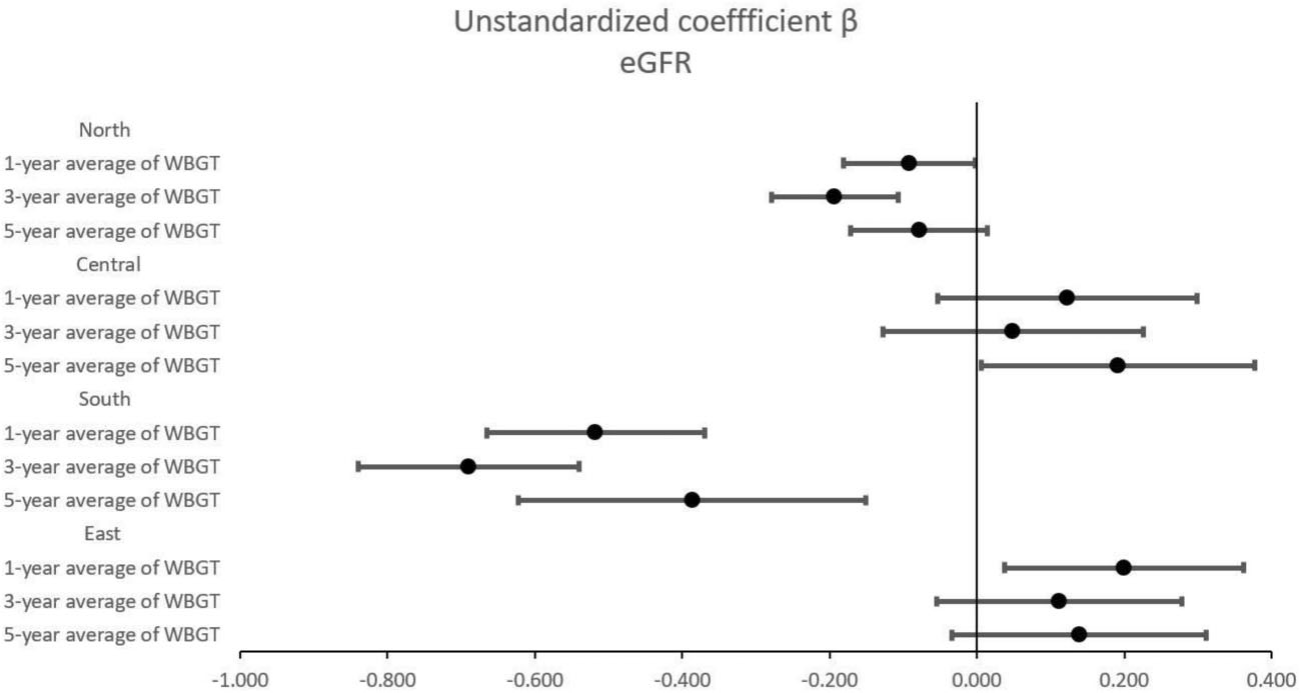
Forest plots of the association of WBGT in the work period with eGFR using multivariable linear regression analysis stratification by geographic regions.

Table 4 shows the associations between WBGT and eGFR in different geographic regions of Taiwan using multivariable linear regression analysis after adjusting for age, sex, smoking and alcohol history, DM, hypertension, BMI, systolic and diastolic BPs, regular exercise, fasting glucose, triglycerides, total cholesterol, HDL cholesterol, LDL cholesterol, haemoglobin and uric acid. In northern Taiwan, during the noon period, the 1, 3 and 5-year average WBGT values per 1°C increase were significantly associated with low eGFR (unstandardized coefficient β = −0.244, *P* < .001; β = −0.399, *P* < .001; and β = −0.234, *P* < .001, respectively). Additionally, during the working period, the 1- and 3-year average WBGT values per 1°C increase were significantly associated with low eGFR (unstandardized coefficient β = −0.092, *P* = .043 for 1 year; β = −0.193, *P* < .001 for 3 years). In southern Taiwan, during the noon period, the 1, 3 and 5-year average WBGT values per 1°C increase were significantly associated with low eGFR (unstandardized coefficient β = −0.493, *P* < .001; β = −0.634, *P* < .001; and β = −0.191, *P* = .162, respectively). In addition, during the working period, the 1-, 3- and 5-year average WBGT values per 1°C increase were significantly associated with low eGFR (unstandardized coefficient β = −0.518, *P* < .001; β = −0.690, *P* < .001; and β = −0.386, *P* = .001, respectively). In eastern Taiwan, during the noon period, the 1-, 3- and 5-year average WBGT values per 1°C increase were significantly associated with low eGFR (unstandardized coefficient β = −0.311, *P* < .001; β = −0.330, *P* < .001; and β = −0.416, *P* < .001, respectively). The associations between WBGT and eGFR during the noon and working periods at 1, 3 and 5 years in different geographic regions of Taiwan are presented in Figs. 2 and 3.

**Table 4:** Association of WBGT with eGFR in different geographic regions using multivariable linear regression analysis.

Average of WBGT (per 1°C)	eGFR (multivariable), unstandardized coefficient β (95% CI)	*P*-value
Northern Taiwan		
Noon period		
1-year	−0.244 (−0.366 to −0.122)	<.001
3-year	−0.399 (−0.516 to −0.283)	<.001
5-year	−0.234 (−0.360 to −0.107)	<.001
Work period		
1-year	−0.092 (−0.181 to −0.003)	.043
3-year	−0.193 (−0.279 to −0.107)	<.001
5-year	−0.079 (−0.171–0.014)	.096
Central Taiwan		
Noon period		
1-year	−0.057 (−0.216–0.101)	.478
3-year	−0.096 (−0.260–0.069)	.254
5-year	0.100 (−0.084–0.283)	.287
Work period		
1-year	0.123 (−0.053–0.298)	.170
3-year	0.049 (−0.128–0.225)	.590
5-year	0.192 (0.005–0.378)	.044
Southern Taiwan		
Noon period		
1-year	−0.493 (−0.656 to −0.329)	<.001
3-year	−0.634 (−0.804 to −0.465)	<.001
5-year	−0.191 (−0.459–0.077)	.162
Work period		
1-year	−0.518 (−0.665 to −0.370)	<.001
3-year	−0.690 (−0.839 to −0.540)	<.001
5-year	−0.386 (−0.623 to −0.150)	.001
Eastern Taiwan		
Noon period		
1-year	−0.311 (−0.492 to −0.130)	<.001
3-year	−0.330 (−0.491 to −0.169)	<.001
5-year	−0.416 (−0.594 to −0.238)	<.001
Work period		
1-year	0.200 (0.037–0.362)	.016
3-year	0.112 (−0.055–0.278)	.189
5-year	0.139 (−0.033–0.311)	.114

Multivariable model: adjusted for age, sex, smoking and alcohol history, diabetes, hypertension, BMI, systolic and diastolic BPs, regular exercise, fasting glucose, triglycerides, total cholesterol, HDL cholesterol, LDL cholesterol, haemoglobin and uric acid.

## DISCUSSION

In this study, we found that increases in the 1-, 3- and 5-year average WBGT values during the noon and working periods were significantly associated with low eGFR. In addition, subgroup analysis by different regions revealed that in southern Taiwan, every 1°C increase in WBGT had a greater impact on the decrease in eGFR compared with other regions in Taiwan.

The first important finding of this study is that the increases in WBGT during both the noon and working periods were significantly associated with low eGFR. A systematic review and meta-analysis in 2021 analysing 42 studies found that a significant linear correlation between ambient temperature and the risk of acute kidney injury (AKI) by 1.2% with every 1°C increase in ambient temperature [18]. In addition, the meta-analysis revealed that the risks of urolithiasis and kidney disease–associated mortality were also significantly increased with increasing temperature [18]. Corroborating this finding, a retrospective study conducted in San Diego also reported a linear correlation between increasing ambient temperatures and an elevated risk ratio of acute renal failure in coastal regions [19]. The consistent observation of a linear relationship across the meta-analysis and the individual retrospective study highlights the potential impact of rising ambient temperatures on AKI.

Previous research has observed that recurrent AKI increases the likelihood of progression to chronic kidney disease (CKD) [20], and this could be a potential pathway through which heat stress contributes to the development of CKD. Mesoamerican nephropathy, an epidemic of CKD of unknown aetiology among sugarcane workers in Central America, is considered a type of heat stress nephropathy related to recurrent dehydration and heat stress [7]. This may also explain the findings of our study. Under conditions of heat exposure and dehydration, glucose is converted to sorbitol via the polyol–fructokinase pathway with the activation of aldose reductase in proximal tubules to protect renal medullary cells against the hypertonic environment [21]. However, under conditions of recurrent heat stress and dehydration, sorbitol is converted into fructose, which activates the fructokinase pathway. This leads to a decrease in adenosine triphosphate availability and an increase in oxidative stress and inflammation, as well as the production of uric acid in proximal tubules, ultimately causing kidney injury [21]. Nevertheless, heat stress–related AKI results not only from dehydration and reduced renal blood flow, but also from tubular damage caused directly by heat stress, as well as the subsequent inflammatory immune response [22]. Increased neutrophils and bone marrow–derived macrophage infiltration into the kidneys, which is associated with exacerbations of tubular damage and fibrosis, have been observed as early as 24 hours after heat stress exposure [22]. In contrast, a decrease in the number of tissue-resident macrophages, which play important roles in the resolution of inflammation and tissue repair, has also been reported after exposure to heat stress. In addition, kidney macrophages polarize to the pro-inflammatory M1 phenotype immediately after exposure to heat stress, which exacerbates inflammation [22]. This phenomenon where temperature influences kidney function was observed not only in hot regions, but also in relatively cooler areas. In the subtropical climate of Taipei, temperature was significantly associated with CKD, with the lowest risk of emergency department visits occurring at 18°C. Furthermore, the cumulative 4-day relative risk (RR) for emergency department visits related to CKD was significant (RR 2.36) at 32°C [23]. On the other hand, in the temperate climate of Adelaide, Australia, a study showed that a 1°C increase in daily minimum temperature was associated with an increase in daily emergency department visits for renal failure [incidence rate ratio (IRR) 1.03] and CKD (IRR 1.02) [24].

The second important finding of this study is that in southern Taiwan, every 1°C increase in WBGT had a greater impact on the decrease in eGFR compared with other regions in Taiwan. To our knowledge, this is the first study to identify this phenomenon. Statistics from the Executive Yuan of Taiwan for the first half of 2021, based on our geographical categorization method, showed that more people are employed in agriculture, forestry, fishing and animal husbandry in the southern part of Taiwan than in other areas [25]. Compared with other industries, these jobs require long hours of labour in humid and hot environments. Although there is no explicit research, the consumption of fructose-sweetened beverages may be higher due to sweating and physically demanding work. As mentioned earlier, the polyol–fructokinase pathway plays a significant role in kidney injury during heat stress. Consuming fructose-sweetened beverages while dehydrated during exercise or labour in hot environments can further elevate the risk of kidney injury [21]. Furthermore, heat stress reduces renal blood flow and GFR, but when combined with dehydration or heavy exercise, it can further decrease renal blood flow and GFR [21]. Interestingly, although fluid replacement can alleviate these effects, compared with drinking water, reducing the increase in core temperature through upper body skin cooling is more effective in mitigating kidney function decline [26]. GFR decreases during moderate and heavy exercise, a process involving renal sympathetic nerve activity, leading to renal vasoconstriction [27]. The mechanism by which heat stress combined with exercise causes a greater decline in kidney function is currently unclear, but it may be due to a decrease in renal blood flow [26]. Exercise and heat stress together have synergistic effects on the release of vasopressin, without involving angiotensin II [26]. This is supported by previous studies indicating that the use of angiotensin-converting enzyme inhibitors does not affect creatinine clearance during exercise in high temperatures [28]. The mechanisms mentioned above may partially explain why the increase in WBGT in southern Taiwan has a more pronounced effect on the decrease in eGFR.

The third important finding of this study is that in eastern Taiwan, the WBGT during the noon period was significantly negatively associated with eGFR, but the WBGT during the work period was positively associated with eGFR. Taiwan's eastern region has large differences in altitude. Past research has indicated that aboriginal people and a lack of healthcare resources are both risk factors for impaired renal function [2]. Therefore, we hypothesize that aboriginal people residing in high-altitude mountainous areas with insufficient healthcare resources have relatively lower eGFRs compared with people in urban flatland areas. In high-altitude mountain regions, temperatures drop more rapidly outside of the noon hours, resulting in a larger difference between the work period WBGT and noon period WBGT. Statistically, this causes the relationship between WBGT and eGFR to transition from a negative correlation during the noon period to a positive correlation during the work period.

The main strengths of this study are the large study cohort and comprehensive data on WBGT. However, there are several limitations that should also be considered. First, this is a cross-sectional study, so we could not evaluate the relationship between WBGT and kidney function over time or the incidence of CKD. We also could not investigate how seasonal changes affect kidney function. Further studies are required to validate our results. Second, the study populations were relatively healthier compared with the general population, which may have affected the association between WBGT and eGFR. Nevertheless, we still identified significant correlations, which may alert physicians to the effects of temperature on renal function. Third, we only estimated outdoor WBGT, and we lacked information on indoor WBGT. This could have introduced some degree of error in the assessment of WBGT exposure. When outdoor temperatures are high, the use of air conditioners and dehumidifiers can reduce the occurrence of extreme heat and humidity indoors, protecting those inside from exposure to high-temperature and high-humidity environments. Therefore, we hypothesize that the use of air conditioners and dehumidifiers would lead to an underestimation of the relationship between WBGT and renal injury. However, previous studies have reported a high correlation between indoor and outdoor temperatures when the outdoor temperature is >12.7°C [29]. This correlation may also be extended to WBGT, providing credibility to our approach of applying outdoor WBGT estimates to indoor environments. Finally, the TWB collects health-related data on healthy volunteers across Taiwan, and women may be more willing or able to participate in research studies compared with men due to greater health awareness. Thus our findings may not be generalizable to the general population.

## CONCLUSIONS

In conclusion, our results revealed that increases in 1-, 3- and 5-year average WBGT values during both the noon and working periods were associated with low eGFRs in Taiwanese adults. Moreover, we observed that in southern Taiwan, every 1°C increase in WBGT had a greater impact on the decrease in eGFR compared with other regions in Taiwan. These findings highlight the importance of heat protection for people working in outdoor or high-temperature environments, as a measure to prevent negative impacts on kidney function. By supporting the development of policies to improve the working conditions of these high-risk groups, coupled with education on the importance of frequent hydration, this study could help reduce the impact of heat stress on their kidney function.

## Data Availability

The data underlying this study are from the Taiwan Biobank and Taiwan Air Quality Monitoring Database. Due to restrictions placed on the data by the Personal Information Protection Act of Taiwan, the minimal dataset cannot be made publicly available. Data may be available upon request to the corresponding author.
